# Influence of Pre-Service Training on STEM Teachers’ Attitudes Toward ICT-Enhanced Teaching: Mediating Roles of Perceived Ease of Use and Perceived Usefulness

**DOI:** 10.3390/bs15101328

**Published:** 2025-09-28

**Authors:** Yingqian Zhang, Jiabin Zhu

**Affiliations:** School of Education, Shanghai Jiao Tong University, Shanghai 200240, China; jiabinzhu@sjtu.edu.cn

**Keywords:** STEM teacher, attitude, ICT-enhanced teaching, pre-service, training strategies

## Abstract

Integrating information and communication technology (ICT) into STEM education enhances instructional quality and cultivates students’ interdisciplinary problem-solving. STEM teachers’ attitudes—driven by perceived ease of use (PEOU) and perceived usefulness (PU)—are pivotal in ICT adoption, and pre-service training offers a vital opportunity to shape these attitudes. Yet, empirical studies investigating how specific training strategies influence ICT attitudes via PEOU and PU remain scarce. Using a mixed-methods approach combining questionnaires and interviews, the results indicate that pre-service training significantly improved STEM teachers’ attitudes toward ICT-enhanced teaching. Socially interactive strategies (role models and collaboration) enhanced attitudes via PEOU by boosting confidence and reducing technology-related anxiety, cognitive design strategies (reflection and instructional design) operated through PU by emphasizing ICT’s pedagogical value, and experiential feedback strategies (authentic experience and feedback) influenced attitudes through both PEOU and PU, fostering integrated technical and pedagogical development. These findings support an integrated SQD–TAM framework and provide practical guidance for designing pre-service STEM teacher programs to promote sustained ICT adoption in China, and meanwhile highlights the importance of strategically sequencing training to cultivate both technological competence and pedagogical insight among future STEM educators.

## 1. Introduction

The integration of information and communication technology (ICT) into STEM education has become a key strategy for enhancing instructional quality and fostering students’ interdisciplinary problem-solving and innovation skills (Banate & Bauyot, 2025; Barakabitze et al., 2019; K. D. Wang et al., 2025). Rapid advances in technologies such as artificial intelligence, big data analytics, and virtual or augmented reality are reshaping STEM curricula toward digitalization and intelligent design, enriching pedagogical resources and teaching methods while raising expectations for teachers’ technological pedagogical competence. Effective integration, however, requires more than technical proficiency; STEM teachers’ attitudes, beliefs, and confidence play a critical role in whether technology is used meaningfully in instruction (Cullen & Greene, 2011; Ertmer & Ottenbreit-Leftwich, 2010; Pozas et al., 2024; Stinken-Rösner et al., 2023). Given that STEM education inherently emphasizes interdisciplinary, applied, and innovative teaching, STEM teachers occupy a critical role: their pedagogical choices directly influence how technology is embedded in complex learning environments, shaping students’ skills development and preparedness for an increasingly digital world.

The Theory of Planned Behavior (Ajzen, 1991) posits that behavioral intention is shaped by attitude, subjective norms, and perceived behavioral control, with attitude—defined as an individual’s affective evaluation and value judgment of a behavior—serving as a central predictor (Lee et al., 2010). In the context of STEM teaching, a positive attitude reflects recognition of technology’s pedagogical potential and confidence in its classroom application (Ertmer & Ottenbreit-Leftwich, 2010; Teo, 2011). Despite widespread ICT training, many STEM teachers still use technology primarily as a supplementary tool rather than a transformative pedagogical resource (Kırkıç & Arıkan, 2023; Mishra & Koehler, 2006). Limited attitudes and low self-efficacy can constrain both the depth and effectiveness of technology adoption, reducing the potential impact on student learning and engagement (Ertmer & Ottenbreit-Leftwich, 2010; K. D. Wang et al., 2025). This issue is particularly critical in the context of educational informatization—the systematic integration of digital technologies into teaching, learning, and school management—which relies heavily on teachers’ readiness and proactive engagement to realize meaningful improvements in teaching quality and learning outcomes.

Pre-service teacher education offers a critical window for shaping instructional beliefs and attitudes before professional habits are established (Eshete, 2022). Research indicates that well-designed curricula, targeted instructor support, abundant teaching resources, and a positive school culture can nurture favorable attitudes toward technology, enhance self-efficacy, and promote readiness to integrate ICT innovatively (Pozas et al., 2024; Şenyiğit & Serin, 2022; Syahrir et al., 2024). Nonetheless, pre-service STEM teachers often experience technology anxiety and challenges in integrating interdisciplinary content with digital tools, which may undermine the formation of positive attitudes (Barak, 2014; B. Wu et al., 2021). Given the central role of STEM teachers in educational informatization, their competence and confidence in leveraging ICT directly determine the success of digitalized and innovation-oriented STEM instruction.

Research systematically examining how specific pre-service training strategies shape STEM teachers’ attitudes—which are subsequently reflected in early-career STEM teachers’ cognitive, affective, and behavioral engagement with ICT-enhanced teaching—remains limited. Existing studies often focus on technical skill acquisition or general attitudes (Ghavifekr & Rosdy, 2015; Pozas et al., 2024), without elucidating how different strategies influence their understanding, emotional engagement, or intentions to apply ICT in instruction. This gap constrains the development of evidence-based reforms in STEM teacher education aimed at improving their ICT competency.

To address this need, the present study focuses on early-career STEM teachers in junior high school in City H, China, employing a mixed-methods design to statistically examines the pre-service training on early-career STEM teachers’ cognitive, affective, and behavioral dimensions of attitudes toward ICT-enhanced teaching. By elucidating these mechanisms, the study contributes theoretically by linking pre-service training strategies to the multi-dimensional attitude development of STEM teachers, and practically by providing evidence-based insights for designing STEM teacher education programs that cultivate both competence and confidence in ICT integration. Ultimately, the findings aim to enhance STEM teachers’ ICT competence, promote the effective and meaningful integration of technology in STEM education, and inform broader innovations and transformative practices in STEM teaching and learning.

## 2. Literature Review

STEM teachers’ attitudes toward ICT-enhanced teaching—shaped by perceived usefulness, ease of use, and self-efficacy—are critical for meaningful technology integration. The Technology Acceptance Model (TAM) clarifies the underlying cognitive–affective mechanisms, while the Synthesis of Qualitative Evidence (SQD) model identifies concrete strategies for cultivating these attitudes in pre-service contexts. However, limited context-specific evidence and insufficient alignment between training practices and institutional support underscore the need for integrated, theory-informed approaches to strengthen STEM teacher preparation for sustained ICT adoption.

### 2.1. STEM Teachers’ Attitudes Toward ICT Integration

With the ongoing digital transformation in education, teachers’ ICT competency has emerged as a crucial factor for improving teaching quality and fostering students’ interdisciplinary skills. This competence is not limited to technical proficiency; it also encompasses pedagogical adaptation, creative integration, and the ability to scaffold students’ problem-solving and inquiry processes using digital tools (Peng et al., 2024; Rastogi & Malhotra, 2013). In particular, teachers’ attitudes toward ICT teaching play a central role, as they shape both the willingness and approach to integrating technology into classroom practices (Zamir & Thomas, 2019). Attitude, in this context, refers to a relatively stable psychological disposition reflecting evaluative judgments, beliefs, and behavioral tendencies toward ICT integration, which collectively influence instructional choices and strategies (Ajzen, 1991; Akarawang et al., 2015).

In STEM contexts, these attitudes are multi-dimensional, including affective orientations toward technology, value-based judgments on ICT integration, and self-efficacy in technology-supported pedagogy (Hämäläinen et al., 2021). Affective components capture STEM teachers’ emotional responses to using ICT, such as interest, enjoyment, or anxiety, whereas cognitive components reflect STEM teachers’ perceptions of ICT’s utility and relevance to learning objectives (Aydin et al., 2016; Frenzel et al., 2021; Holden & Rada, 2011). Self-efficacy, meanwhile, pertains to confidence in designing, implementing, and managing technology-enhanced lessons (Yasin et al., 2020; Zhao et al., 2025). Together, these cognitive and affective dimensions strongly predict both the likelihood and quality of technology adoption in classrooms, influencing not only whether digital tools are used but also how they are applied to enhance engagement, interactivity, and student-centered learning (Instefjord & Munthe, 2017; Player-Koro, 2012; Martin et al., 2025).

The formation of STEM teachers’ instructional behaviors is closely tied to these attitudes, as attitudes primarily influence behavioral intentions and subsequent actions (Abel et al., 2022; Peng et al., 2024; Turgut & Aslan, 2021). STEM teachers who hold positive attitudes toward ICT are more likely to integrate digital tools proactively, designing lessons that capitalize on technology to support collaboration, problem-solving, and creativity (Adov et al., 2020; Barakabitze et al., 2019). Positive attitudes also encourage sustained professional development, motivating STEM teachers to expand technological literacy and pedagogical strategies over time (Hopcan et al., 2024; Savec, 2019). Conversely, even teachers with favorable attitudes may face challenges: increased technology complexity or insufficient support can trigger anxiety, undermine confidence, and restrict the consistent application of ICT, highlighting that attitude encompasses both willingness and perceived capability (Alieto et al., 2024; Demirtas & Eksioglu, 2020; D. Wu et al., 2022).

In Chinese context, existing studies have mainly focused on defining attitude constructs and developing preliminary measurement tools, often constrained to regional or small-scale samples. For instance, Zhu (2016) identified a direct link between teachers’ cognitive understanding of technology–pedagogy integration and practical ICT use in ISichuan Province, while Y. Tang et al. (2019) emphasized in their investigation in Guangdong that taking attitude and teaching awareness as important components of teachers’ ICT capability structure is helpful for understanding teachers’ teaching behavior tendencies. Xiang et al. (2006) reported that nearly 70% of teachers evaluated ICT integration favorably, and He et al. (2022) found high levels of acceptance among STEM teachers in Beijing and Zhejiang. These studies indicate that cognitive understanding and perceived value of technology are key predictors of classroom ICT adoption.

Despite these insights, significant gaps remain regarding theoretical rigor, measurement validity, and generalizability. Many existing assessments lack well-grounded conceptual frameworks and rely primarily on the Ministry of Education’s ICT competence standards, which emphasize technical criteria but do not fully capture STEM teachers’ cognitive, affective, and efficacy-related attitudes (Ministry of Education of the People’s Republic of China, 2014). This limitation leads to instrument arbitrariness, interpretive ambiguity, and restricted explanatory power. Therefore, a pressing research need is to precisely define STEM teachers’ ICT attitudes within China’s unique educational context, integrating both theoretical grounding and psychometric validation. Developing theory-driven, reliable, and valid measurement tools will enable a more comprehensive understanding of teachers’ attitudes and the mechanisms through which these attitudes influence technology integration in STEM classrooms.

### 2.2. The Technology Acceptance Model (TAM) and STEM Teachers

Building on Ajzen and Fishbein’s (1977) broad definition of attitude as an individual’s evaluation of a specific object, the TAM positions behavior-oriented ICT attitudes as central predictors of technology use intentions and behaviors (Zhang & Aikman, 2007; Zhang et al., 2008). This distinction separates general attitudes toward ICT as a tool from attitudes toward specific ICT-related teaching behaviors, emphasizing the educational context of technology integration (Instefjord & Munthe, 2017; Scherer et al., 2015). By differentiating general perceptions from behavior-specific attitudes, the TAM facilitates a more precise understanding of the psychological drivers that influence STEM teachers’ adoption of technology in instructional practices, enabling researchers and practitioners to better target interventions.

**Figure 1:**
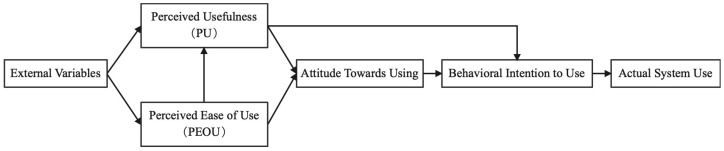
Technology acceptance model (Davis, 1989).

The TAM identifies two core beliefs that shape attitudes: (1) Perceived Usefulness (PU): the belief that technology enhances teaching effectiveness; (2) Perceived Ease of Use (PEOU): the belief that technology is easy to learn and operate (Marangunić & Granić, 2015). These constructs interact to determine teachers’ behavioral intentions, which in turn predict actual technology adoption (Lazim et al., 2021; Venkatesh et al., 2003), as illustrated in Figure 1. Extensive empirical validation across diverse domains, including digital libraries, e-learning, e-health, and educational settings, has confirmed the TAM’s robustness and adaptability (Granić & Marangunić, 2019; Lazim et al., 2021; Palaniswamy & Raj, 2022; Rafique et al., 2020; H. Wang et al., 2022). Nonetheless, meta-analyses indicate that pathway strengths and the influence of contextual and external factors require adaptation to accurately capture the complexities of teaching environments (Scherer et al., 2019).

Applied to STEM teachers, the TAM clarifies how cognitive understanding, subject knowledge depth, and beliefs about ICT form the foundation for PU and PEOU, thereby shaping attitudes toward technology-integrated teaching (Scherer et al., 2015; Sun et al., 2024). These attitudes encompass multiple dimensions—cognitive evaluations, affective responses, and self-efficacy—each critical to the development of technology integration competence (Scherer et al., 2019). Importantly, the TAM not only predicts whether teachers will adopt technology but also explains how perceptions influence attitudes and intentions, highlighting barriers such as perceived complexity or lack of confidence that may inhibit effective use (Pearce, 2022; Scherer et al., 2018; Ateş & Gündüzalp, 2024). This insight provides a theoretical basis for designing valid measurement instruments and targeted professional development programs tailored to teachers’ specific needs.

Furthermore, the TAM offers practical guidance for policymakers, curriculum designers, and teacher educators by elucidating the psychological mechanisms underlying technology acceptance (Luhamya et al., 2017). Integrating the TAM insights into teacher preparation enables simultaneous enhancement of technical proficiency (improving PEOU) and pedagogical understanding of ICT benefits (improving PU), thereby fostering positive attitudes and promoting sustainable integration of technology into classroom practice (Gupta et al., 2021; Sungur Gül & Ateş, 2023).

In summary, the TAM provides a robust, empirically supported theoretical framework for analyzing and promoting STEM teachers’ ICT attitudes and behaviors. By clarifying the causal chain from perceptions to attitudes, intentions, and actual instructional practice, the TAM serves as both a diagnostic tool for understanding current adoption patterns and a guiding framework for designing interventions that enhance teachers’ competence and confidence in technology-integrated teaching.

### 2.3. Pre-Service Training Strategies and Attitude Formation

As educational informatization continues to deepen globally, the competence of STEM teachers in technology-integrated teaching has emerged as a core professional literacy. This competence encompasses not only the technical ability to use digital tools but also the pedagogical knowledge required to embed technology meaningfully into instruction. In particular, pre-service teacher education represents a pivotal period for shaping teachers’ attitudes, value perceptions, and instructional inclinations toward ICT use (Bueno-Alastuey & Villarreal, 2021). During this formative stage, teachers develop foundational beliefs about the usefulness and applicability of technology in enhancing student learning outcomes, as well as the confidence and motivation to implement these tools effectively in classroom settings. These early experiences play a crucial role in determining the trajectory of teachers’ professional development and their long-term engagement with technology-enhanced teaching practices (Göltl et al., 2024; Lausa et al., 2024).

Grounded in the TAM (Davis, 1989), pre-service training influences teachers’ attitudes through interconnected cognitive and affective pathways. Systematic curriculum design coupled with practical teaching opportunities enhances teachers’ technological knowledge and hands-on operational experience, thereby improving both their PU of technology and PEOU (Wasserman & Migdal, 2019). At the same time, reflective learning activities, mentorship, and guided feedback facilitate the internalization of technology use within teachers’ pedagogical beliefs and professional values, deepening their affective commitment and motivation to integrate ICT meaningfully into instruction (Jamissen & Phelps, 2006; Sun et al., 2024). This dual influence underscores that effective attitude formation relies on both the acquisition of technical skills and the cultivation of a positive, value-oriented mindset toward technology (Buabeng-Andoh, 2012; Shahali & Halim, 2024).

Despite growing research attention, many existing studies on pre-service training focus primarily on isolated strategies or discrete interventions, such as single workshops, technology courses, or classroom simulations (Coffey, 2021; Pozas & Letzel, 2023; Yang et al., 2022). Such narrow investigations often fail to capture how multiple strategies interact synergistically over time, or how teachers’ attitudes evolve dynamically across different stages of their pre-service experiences (Tondeur et al., 2018). Moreover, a notable gap exists between micro-level instructional practices—such as classroom simulations, peer collaboration, and hands-on practicums—and macro-level institutional support, including school policies, curriculum frameworks, and organizational guidance (Marbán & Sintema, 2021; Pozas et al., 2024; Zyad, 2016). This disconnect can limit the effectiveness of professional development programs and impede the formation of sustained positive attitudes toward technology integration.

**Figure 2:**
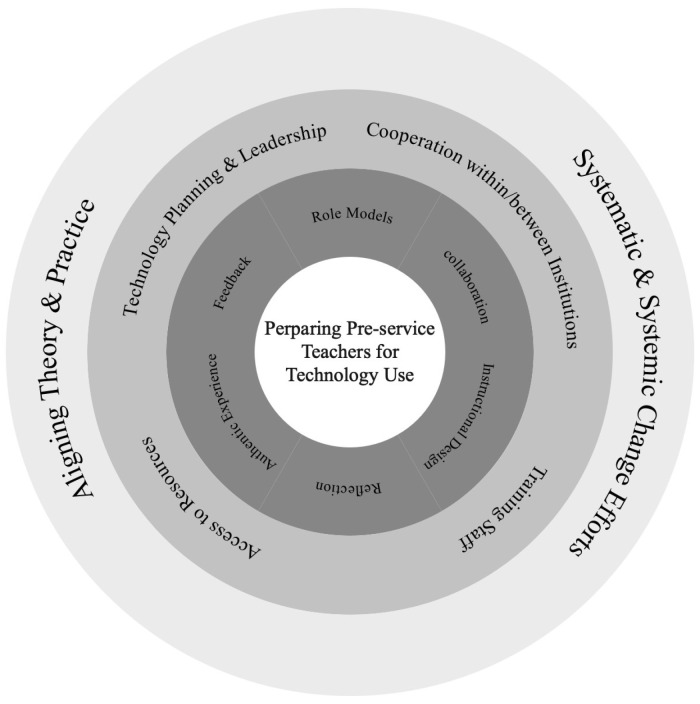
SQD model to prepare pre-service teachers for technology use (Tondeur et al., 2012).

To address these challenges, Tondeur et al. (2012) proposed the SQD model, which provides a structured framework for integrating key pre-service training strategies across policy, organizational, and practice dimensions. At the micro level, the SQD model identifies six essential strategies—role models, collaboration, reflection, instructional design, authentic practice, and continuous feedback—that directly shape teachers’ attitudes by offering rich, authentic, and diversified experiential learning opportunities (see Figure 2) (Knezek et al., 2023). The integration of the SQD model with the TAM bridges practical training and psychological theory: the SQD model guides actionable, contextually grounded interventions, while the TAM explicates the perception–attitude–behavior causal pathways through which these interventions foster the development of stable, positive attitudes toward ICT (Galimova et al., 2024; Hsu & Lin, 2020; Kukul, 2023; Pan et al., 2025).

Empirical applications of the SQD model in China have provided early evidence of its effectiveness. C. Tang et al. (2019) demonstrated that combining peer evaluation, expert lectures, and practicum-based activities significantly enhanced pre-service STEM teachers’ ICT literacy and fostered more favorable attitudes toward technology integration. In contrast, research by Miao et al. (2019) highlighted that insufficient opportunities for authentic practice and structured reflection were key barriers limiting the development of positive attitudes and reducing teachers’ willingness to implement ICT in classroom teaching. These findings collectively emphasize the importance of balancing knowledge acquisition with substantial hands-on practice and continuous reflective support to cultivate enduring positive attitudes.

Overall, the micro-level strategies provide a comprehensive and dynamic approach to fostering STEM teachers’ ICT perceptions, affective identification, and instructional willingness (Tondeur et al., 2012). By systematically addressing both technical and affective dimensions of competence, these strategies lay the foundation for stable, positive attitudes, which are essential for the development of robust technology integration skills (Tondeur et al., 2018). Building on the original SQD model, Tondeur et al. (2025) further reviewed and synthesized existing literature and proposed the SQD2 model to better account for contemporary developments in teacher education. The SQD2 framework elaborates the original strategy components into more detailed elements, including role models, exploration of emerging technologies, reflective practice, collaborative activities, instructional design, authentic experiences, and structured feedback, highlighting their interactions and providing actionable guidance for pre-service teacher training (Tondeur et al., 2025).

Compared with the original SQD model, SQD2 places greater emphasis on the interplay among strategies, offering a nuanced approach for integrating ICT competence and teaching confidence. In contrast, the present study focuses primarily on the original SQD framework, examining the impact of more fundamental and relatively independent strategies on STEM teachers’ attitudes toward ICT-enhanced teaching. This approach lays a solid foundation for future research investigating the combined and interactive effects of integrated strategies.

### 2.4. Conceptual Framework and Research Hypotheses

**Figure 3:**
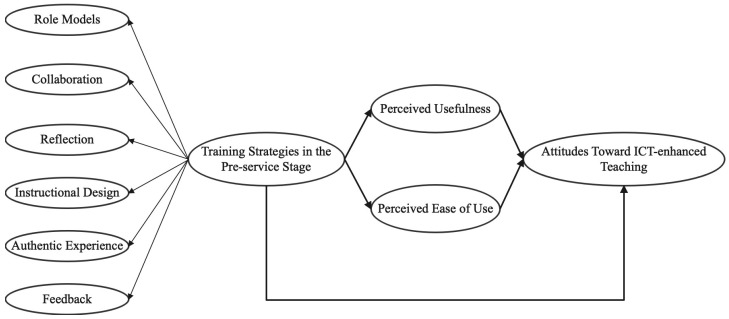
Conceptual framework.

Based on the preceding analysis and identified research gaps, the present study integrates the TAM and SQD frameworks to examine how six micro-level SQD strategies influence early-career STEM teachers’ attitudes toward ICT-enhanced teaching (Figure 3). These strategies are hypothesized to exert direct effects on attitudes and indirect effects via perceived usefulness (PU) and perceived ease of use (PEOU), enabling a systematic investigation of the mechanisms through which pre-service preparation shapes teachers’ willingness, confidence, and competence in ICT integration.

Based on this framework, the study proposes the following research hypotheses:

Role models (ROL) positively influence early-career STEM teachers’ attitudes toward ICT-enhanced teaching **(H1a)**. PU and PEOU are hypothesized to mediate this relationship **(H1b, H1c)**.

Collaboration (COL) positively influences ICT attitudes, with PU and PEOU as mediators **(H2a–H2c)**.

Reflection (REF) positively influences ICT attitudes, with PU and PEOU as mediators **(H3a–H3c)**.

Instructional Design (DES) positively influences ICT attitudes, with PU and PEOU as mediators **(H4a–H4c)**.

Authentic Experience (AUT) positively influences ICT attitudes, with PU and PEOU as mediators **(H5a–H5c)**.

Feedback (FEE) positively influences ICT attitudes, with PU and PEOU as mediators **(H6a–H6c)**.

## 3. Research Method

A mixed-methods design was employed, combining surveys to quantify STEM teachers’ attitudes toward ICT-enhanced teaching and the implementation of pre-service training strategies, with semi-structured interviews to provide qualitative insights. Quantitative data were analyzed using SPSS, while qualitative findings were used to triangulate and enrich the interpretation of the results.

### 3.1. Participants

This study involved early-career STEM teachers from junior high schools in a district of H City, China, covering science, mathematics, information technology, and engineering-related courses. In this context, the “science” curriculum integrates physics, chemistry, biology, geography, and engineering, forming a core disciplinary cluster together with mathematics and information technology. Teachers in these subjects typically demonstrate strong interdisciplinary competencies and practice-oriented characteristics, consistent with the attributes of STEM educators.

According to district government regulations and school-level induction policies, teachers with fewer than five years of teaching experience are classified as “early-career.” The selection of STEM teachers was based on their higher reliance on ICT, which serves not only as a major teaching tool but also as part of their instructional content. Thus, focusing on this group ensures both representativeness and relevance for examining the impact of pre-service training strategies on ICT teaching attitudes.

A total of 49 STEM teachers participated in the interviews. Additionally, 323 questionnaires were distributed, of which 309 were returned as valid, yielding an effective response rate of 95.67%. The sample included 146 male teachers (47.25%) and 163 female teachers (52.75%). Regarding subject distribution, there were 99 mathematics teachers, 144 science teachers, 53 information technology teachers, and 13 dedicated engineering teachers. With respect to grade levels, 117 teachers taught grade 7, 125 taught grade 8, and 67 taught grade 9. The distribution of gender, subject specialization, and grade levels closely reflected the overall characteristics of junior high STEM teachers in the district, supporting the representativeness of the sample for subsequent analyses.

### 3.2. Data Collection

(1) Quantitative Data Collection. Questionnaires were administered online (“Wenjuanxing”) and offline (district-level teaching and research meetings). The instrument comprised three sections: (1) STEM teachers’ attitudes toward ICT-enhanced teaching, adapted from Scherer et al. (2018) and based on TAM, covering general attitudes, perceived usefulness, and perceived ease of use; (2) pre-service training strategies, adapted from the SQD model (Tondeur et al., 2016), assessing six training strategies of ICT-related preparation; and (3) demographic and professional background. The questionnaire items were translated and culturally adapted to the Chinese context, with adjustments to phrasing and item order. To ensure content validity, the instrument was reviewed by university faculty, practicing secondary STEM teachers, and researchers. Items employed a five-point Likert scale, with demographic data collected categorically or open-ended. Both scales demonstrated satisfactory reliability and validity (ICT attitudes: Cronbach’s α = 0.763, KMO = 0.787; training strategies: Cronbach’s α = 0.890, KMO = 0.852), ensuring their suitability for quantitative analysis. The questionnaire with all items is detailed in Appendix A.

(2) Qualitative Data Collection. Semi-structured interviews were conducted to complement and interpret the survey results, with a focus on uncovering the internal mechanisms through which pre-service training strategies shape early-career STEM teachers’ attitudes toward ICT-enhanced teaching and their subsequent development. Guiding questions explored STEM teachers’ perceptions of ICT use, adoption of new tools, and personal interest in technology. Interviews were conducted both online and offline, recorded with informed consent, and transcribed verbatim for coding and thematic analysis.

### 3.3. Data Analysis

Quantitative analyses were conducted in SPSS 26.0 to examine the relationships in the conceptual framework (Figure 3). Descriptive statistics characterized STEM teachers’ attitudes and pre-service training implementation, and reliability and validity were assessed via Cronbach’s α and KMO. To explore the differential effects of pre-service training strategies on STEM teachers’ attitudes and their mediating mechanisms, mediation analyses were performed using the PROCESS macro. PROCESS was selected for its flexibility in testing complex mediation models, its ability to handle multiple mediators simultaneously, and its robust bootstrap-based estimation of indirect effects, which is particularly suitable for exploratory studies with moderate sample sizes (Abu-Bader & Jones, 2021). Its widespread use in educational technology and teacher education research also ensures comparability with prior studies, making it well-aligned with the study’s focus on identifying nuanced mechanisms linking training strategies to early-career STEM teachers’ attitudes.

Qualitative data were analyzed in ATLAS.ti following Braun and Clarke’s six-phase thematic analysis. Double-coding and iterative comparison of representative text excerpts facilitated the identification of key mechanisms linking pre-service training strategies to early-career STEM teachers’ ICT competencies (Braun & Clarke, 2019).

## 4. Result

### 4.1. STEM Teachers’ Attitude Towards ICT-Enhanced Teaching

The study assessed early-career STEM teachers’ attitudes toward ICT-enhanced teaching, focusing on perceived usefulness (PU), perceived ease of use (PEOU), and general attitudes toward ICT-enhanced teaching (ICT-ATT). Descriptive statistics for each dimension are summarized in Table 1. The results indicate that the mean scores for all three dimensions exceeded 4.00 on a five-point Likert scale, reflecting a generally positive disposition among teachers toward ICT-enhanced instruction.

These findings indicate that early-career STEM teachers generally recognize the benefits of digital technologies for enhancing teaching effectiveness and classroom engagement, while also perceiving ICT as relatively easy to use. Their positive attitudes encompass both cognitive evaluations—such as perceived relevance and instructional value—and affective components, including confidence and motivation to integrate ICT meaningfully. This readiness provides a foundation for examining how pre-service training strategies may further shape attitudes and support consistent ICT use, and it can also guide the design of targeted interventions and professional development programs to sustain effective technology integration among early-career STEM educators. Supporting this, one participant stated: “I believe that ICT is essential for modern teaching. It not only improves teaching quality and effectiveness but also stimulates students’ interest in learning. Therefore, I continually strive to learn and explore, hoping these efforts will enhance my professional skills in ICT teaching and benefit my students.” (SC18)

### 4.2. Implementation of Pre-Service Training Strategies

Descriptive statistics (Table 2) show that mean scores for six pre-service training strategies—role models, collaboration, reflection, instructional design, authentic experience, and feedback—exceeded 4.00 on a five-point Likert scale, indicating that teachers generally perceived themselves as well-prepared for ICT-enhanced instruction.

Specifically, teachers reported that role models and collaborative activities were highly effective in providing guidance and peer support, while reflection and instructional design facilitated the integration of ICT into lesson planning and pedagogical practice. Authentic experiences, such as teaching practicums, were rated slightly higher than other strategies, highlighting the value of hands-on opportunities in consolidating technological skills and confidence. Continuous feedback also demonstrated a positive influence, although it exhibited the widest range, suggesting variability in how feedback was delivered and utilized across training contexts. One participant highly valued the pre-service practical sessions, stating: “These were primarily hands-on activities. We worked in groups to practice creating instructional materials or simulate classroom teaching, including using tools such as clickers for interaction. After completing the exercises, we provided feedback to one another, and instructors also offered professional evaluations and suggestions to help us improve.” (SC18)

These results collectively suggest that pre-service programs in H City have successfully implemented comprehensive ICT-related training strategies, supporting early-career STEM teachers in developing both the knowledge and the practical skills necessary for technology integration. The generally high ratings across all strategies reflect a well-rounded approach, combining cognitive, affective, and experiential components to foster teachers’ readiness and willingness to incorporate ICT into STEM classrooms.

### 4.3. The Correlation Between Pre-Service Training Strategies and STEM Teachers’ Attitude

Table 3 presents the correlations between pre-service training strategies and early-career STEM teachers’ attitudes toward ICT-enhanced teaching. All six strategies—role models (r = 0.478 **), collaboration (r = 0.513 **), reflection (r = 0.467 **), instructional design (r = 0.513 **), authentic experience (r = 0.399 **), and feedback (r = 0.399 **)—are positively associated with teachers’ general attitude toward ICT (ICT-ATT). Role models and collaboration show the strongest relationships, highlighting the importance of guidance and peer-supported activities. These strategies are also positively correlated with PU PEOU, suggesting that pre-service training may foster positive attitudes by enhancing teachers’ confidence and perceived value of ICT.

### 4.4. Mediating Effect of Perceived Ease of Use and Perceived Usefulness

(1) The mediating effect of PEOU and PU on the relationship between ROL and ICT-ATT

**Figure 4:**
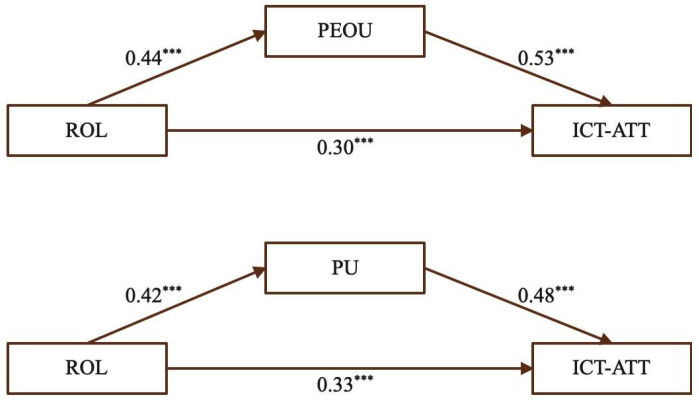
The mediating effect of PEOU and PU for ROL. (Note: *** *p* < 0.001.)

A simple mediation model was constructed with role models as the independent variable, Perceived Ease of Use (PEOU) and Perceived Usefulness (PU) as mediators, and Teachers’ Attitudes Toward ICT-enhanced Teaching (ICT-ATT) as the dependent variable, to examine the underlying mechanism between role models and early-career STEM teachers’ attitudes toward ICT-enhanced teaching. All variables were standardized prior to analysis, the results are illustrated in Figure 4. The analysis indicated that ROL significantly and positively predicted both PEOU and PU, as well as ICT-ATT. PEOU and PU, in turn, significantly and positively predicted ICT-ATT, suggesting that STEM teachers’ cognitive evaluations serve as key channels through which ROL influences ICT-ATT.

To verify the mediating effects, the Bootstrap method was employed, with significance determined by a 95% CI excluding zero. As shown in Table 4, both mediation paths were significant: PEOU mediated the ROL–ICT-ATT relationship with an effect size of 0.23 (95% CI = [0.17, 0.31]), accounting for 43.40% of the total effect, while PU mediated the same relationship with an effect size of 0.20 (95% CI = [0.14, 0.27]), accounting for 37.74% of the total effect. These results support H1a (ROL → ICT-ATT), H1b (ROL → PEOU → ICT-ATT), and H1c (ROL → PU → ICT-ATT).

These results suggest that the role models not only exert a substantial direct positive influence on STEM teachers’ attitudes toward ICT-enhanced teaching, but also indirectly fosters these attitudes by enhancing teachers’ perceptions of both the ease of use and the usefulness of technology. In other words, the presence of effective role models in pre-service training can simultaneously strengthen teachers’ confidence in operating ICT tools and deepen their recognition of the pedagogical benefits such tools offer in STEM instruction. This quantitative finding is supported by qualitative evidence, as one early-career teacher noted during the interviews: “In such training, if experienced teachers or exemplary instructors—or even peers who perform very well—are providing guidance, we are definitely willing to learn. If it is something we need, everyone is very motivated to participate.” (SC17) This statement illustrates how exposure to competent role models can motivate teachers to engage actively with ICT, reinforcing both confidence and perceived pedagogical value.

This dual impact underscores the importance of integrating visible, high-quality role models into teacher preparation programs, as it helps bridge the gap between technical proficiency and meaningful instructional application, ultimately supporting sustainable and effective technology integration in classrooms.

(2) The mediating effect of PEOU and PU on the relationship between COL and ICT-ATT

**Figure 5:**
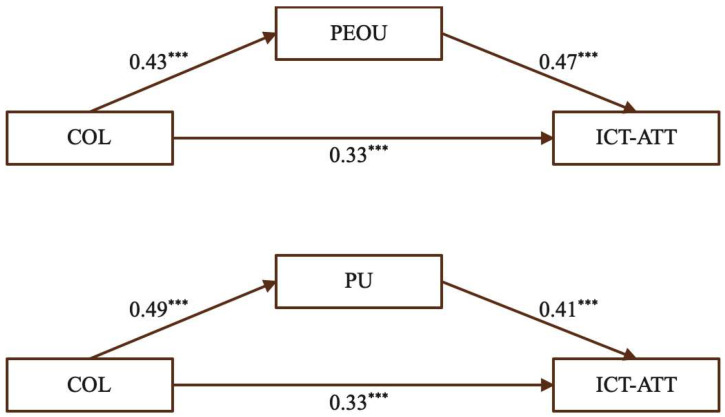
The mediating effect of PEOU and PU for COL. (Note: *** *p* < 0.001.)

Figure 5 illustrates that COL had a significant positive effect on ICT-ATT, and also significantly predicted both PEOU and PU. In turn, PEOU and PU each exerted significant positive effects on ICT-ATT, indicating that both perceived ease of use and perceived usefulness serve as important pathways linking COL to ICT-ATT.

Table 5 presents the mediating roles of PEOU and PU in the effect of collaboration on ICT-ATT. For PEOU, the total effect of COL was 0.53, with a direct effect of 0.33 (62.26%) and an indirect effect of 0.20 (37.74%). For PU, the total effect was 0.53, comprising a direct effect of 0.33 (62.26%) and an indirect effect of 0.20 (37.74%). The statistical significance of the indirect pathways confirms that both mediators meaningfully transmit the effect of collaboration, supporting Hypotheses H2a, H2b, and H2c.

Collaborative experiences among pre-service teachers can foster both technical ease (PEOU) and recognition of ICT’s instructional value (PU). Engaging in collaborative activities enables teachers to exchange practical strategies and problem-solving approaches, promoting confidence in tool usage and awareness of pedagogical benefits. This quantitative insight is reinforced by qualitative evidence from an interviewee, who noted: “We work in groups to practice creating instructional materials or simulate classroom teaching, including using tools like clickers for interaction. After completing these tasks, we provide feedback to each other, and teachers offer professional evaluation and suggestions to help us improve.” (SC11) This statement illustrates how peer collaboration contributes to the development of positive ICT teaching attitudes. Together, these findings underscore the importance of embedding collaborative learning opportunities into training programs to support effective and sustainable integration of ICT in STEM instruction.

(3) The mediating effect of PEOU and PU on the relationship between REF and ICT-ATT

**Figure 6:**
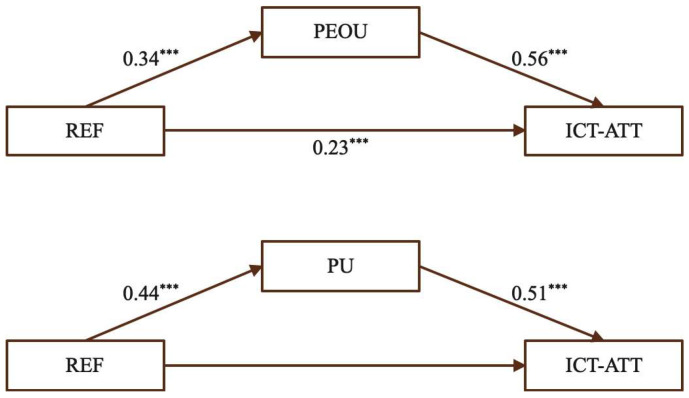
The mediating effect of PEOU and PU for REF. (Note: *** *p* < 0.001.)

Figure 6 shows that REF positively and significantly influenced ICT-ATT, and also exhibited significant positive associations with both PEOU and PU. Likewise, PEOU and PU were found to be significant positive predictors of ICT-ATT. These findings suggest that reflective practice contributes to shaping teachers’ attitudes toward ICT-enhanced teaching through both the perceived ease of use and the perceived usefulness of technology.

As shown in Table 6, for PEOU, the total effect of REF on ICT-ATT was 0.42, with a direct effect of 0.23 (54.76%) and an indirect effect of 0.19 (45.24%). For PU, the total effect was 0.42, including a direct effect of 0.20 (47.63%) and an indirect effect of 0.22 (52.38%). These findings confirm that both PEOU and PU mediate the relationship between reflective practice and ICT-ATT, supporting Hypotheses H3a, H3b, and H3c.

Engaging in reflective practice allows teachers to simultaneously strengthen their confidence in managing ICT tools (PEOU) and enhance their understanding of the pedagogical benefits of technology (PU). Reflective activities help teachers critically evaluate and refine instructional strategies, reinforcing both operational skills and instructional insight. This is supported by qualitative evidence from a participant (TH7), who remarked: “The training not only improved our skills, but more importantly led to deeper shifts in our thinking. Through the program, we reflected on whether our actions were effective, considered how to improve in the future, and began to realize that being a teacher involves not only mastering technology but also thinking about how to integrate it meaningfully into daily instruction. Such experiences have been very helpful as I started my teaching career.” This statement illustrates how structured reflection in pre-service programs fosters a balanced development of technical proficiency and pedagogical understanding, supporting sustainable and effective ICT integration in STEM classrooms.

(4) The mediating effect of PEOU and PU on the relationship between DES and ICT-ATT

**Figure 7:**
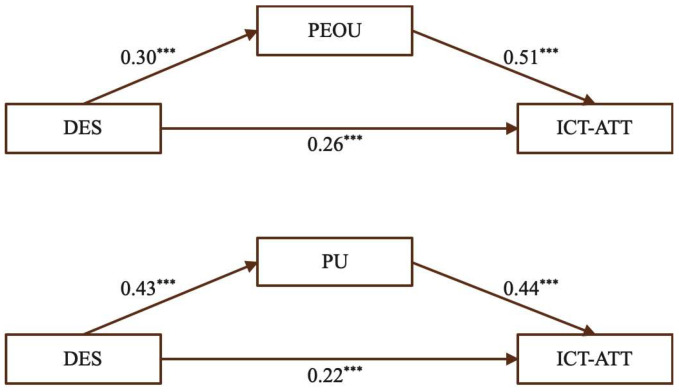
The mediating effect of PEOU and PU for DES. (Note: *** *p* < 0.001.)

Figure 7 shows that DES positively and significantly influenced ICT-ATT, and also significantly predicted both PEOU and PU. In turn, PEOU and PU were significant positive predictors of ICT-ATT, indicating that carefully structured instructional design can shape teachers’ attitudes toward ICT-enhanced teaching through both the perceived ease of use and perceived usefulness of technology.

Table 7 reports the mediating effects of PEOU and PU in the association between instructional design and ICT-ATT. For PEOU, the total effect was 0.41, with a direct effect of 0.26 (63.41%) and an indirect effect of 0.15 (36.59%). For PU, the total effect was 0.41, with a direct effect of 0.22 (53.66%) and an indirect effect of 0.19 (46.34%). The significance of the indirect effects demonstrates that both PEOU and PU serve as meaningful mediators, providing support for Hypotheses H4a, H4b, and H4c.

Thoughtfully structured instructional design can concurrently improve teachers’ confidence in using ICT tools (PEOU) and deepen their awareness of the pedagogical value these tools provide (PU). By engaging with well-planned instructional activities, teachers develop both operational skills and instructional insight. A participant described: “We learned how to use new technologies to enrich our teaching content, such as employing multimedia materials to present traditional mathematical concepts and formulas. We also used animations or simulated experiments to explain complex principles, making them simpler, more vivid, and engaging. In addition, we learned how to design interactive mathematics activities, such as online quizzes and discussions, to enhance students’ engagement and interest.” (MT1) Such experiences illustrate how structured instructional design in pre-service programs fosters the dual development of technical proficiency and pedagogical understanding, highlighting its importance for effective and sustainable ICT integration in STEM classrooms.

(5) The mediating effect of PEOU and PU on the relationship between AUT and ICT-ATT

**Figure 8:**
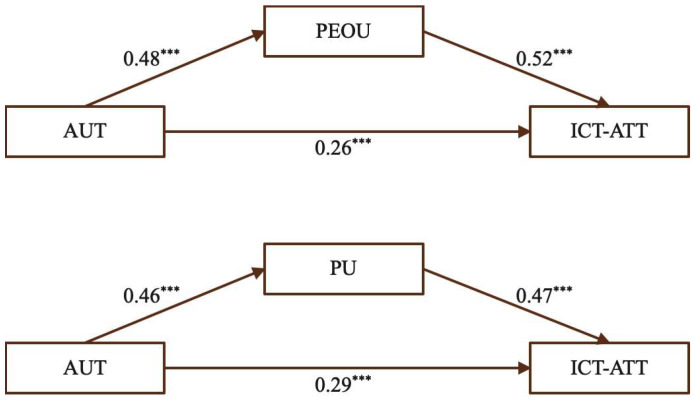
The mediating effect of PEOU and PU for AUT. (Note: *** *p* < 0.001.)

Figure 8 shows that AUT had a significant positive impact on ICT-ATT and was also positively associated with both PEOU and PU. Similarly, PEOU and PU were significant predictors of ICT-ATT, suggesting that engaging in authentic experiences, such as internships, supports the development of favorable attitudes toward ICT-enhanced teaching through both perceived ease of use and perceived usefulness.

According to Table 8, for PEOU, the total effect of AUT on ICT-ATT was 0.51, comprising a direct effect of 0.26 (50.98%) and an indirect effect of 0.25 (49.02%). For PU, the total effect was 0.51, with a direct effect of 0.29 (56.86%) and an indirect effect of 0.22 (43.14%). The indirect effects for both mediators were statistically significant, confirming their roles in transmitting the impact of authentic experiences and supporting Hypotheses H5a, H5b, and H5c.

Participation in authentic experiences, such as internships, can simultaneously enhance teachers’ confidence in applying ICT tools (PEOU) and broaden their understanding of the pedagogical benefits (PU). Practical, real-world engagement allows teachers to connect theory with classroom practice, demonstrating the value of embedding authentic, hands-on experiences in teacher preparation programs to support both technical proficiency and pedagogical insight for sustained ICT integration. This is exemplified by one participant (MT3), who stated: “Only through actual practice can we truly master ICT tools. For instance, knowing that PowerPoint can be used for teaching is one thing, but being able to skillfully create engaging mathematics teaching PPTs is another. Practical training allows us to transform what we have learned into real educational capabilities, anticipate potential problems, and learn how to solve them.”

(6) The mediating effect of PEOU and PU on the relationship between FEE and ICT-ATT

**Figure 9:**
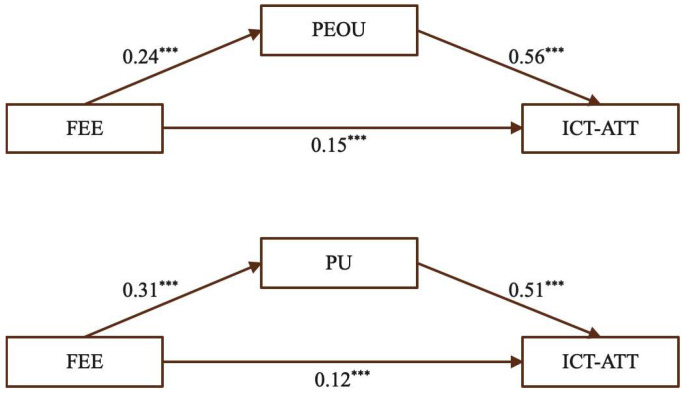
The mediating effect of PEOU and PU. (Note: *** *p* < 0.001.)

Figure 9 shows that FEE positively and significantly influenced ICT-ATT, and also significantly predicted both PEOU and PU. Similarly, PEOU and PU were significant predictors of ICT-ATT, indicating that receiving constructive feedback can shape teachers’ attitudes toward ICT-enhanced teaching through both perceived ease of use and perceived usefulness.

Table 9 presents the mediating effects of PEOU and PU in the relationship between FEE and ICT-ATT. For PEOU, the total effect was 0.28, with a direct effect of 0.15 (53.57%) and an indirect effect of 0.13 (46.43%). For PU, the total effect was 0.28, including a direct effect of 0.12 (53.57%) and an indirect effect of 0.16 (46.43%). The significance of these indirect effects indicates that both PEOU and PU function as effective mediators, providing empirical support for Hypotheses H6a, H6b, and H6c.

Receiving constructive feedback can both strengthen teachers’ confidence in using ICT tools (PEOU) and heighten their appreciation of the instructional benefits of technology (PU). Feedback offers timely guidance that helps teachers refine their practice, improve instructional materials, and consider strategies to engage students more effectively. As one participant stated, “We learned how to use the online learning platform; the teacher had us register in a virtual classroom where we simulated assigning homework, submitting assignments, and conducting online discussions. The teacher guided us throughout, addressing any questions or areas for improvement on the spot. I found this approach very effective.” (MT4) Regarding peer feedback, another participant noted, “During practical exercises, we were divided into groups to interact and learn from each other. Each person had to create instructional materials and assignments, and then we discussed and suggested improvements together.” (SC13) These experiences underscore the importance of incorporating systematic feedback mechanisms into pre-service training to promote enduring and effective ICT integration in STEM classrooms.

## 5. Discussion

This study examined the effects of six pre-service training strategies—Role Models, Collaboration, Reflection, Instructional Design, Authentic Experiences, and Feedback—on early-career STEM teachers’ attitudes toward ICT-enhanced teaching, with Perceived Ease of Use and Perceived Usefulness as mediators. Results indicate that all six strategies exert both direct and indirect influences on teachers’ attitudes, providing a context-specific empirical demonstration of how SQD-based training shapes the cognitive and affective dimensions of technology integration in STEM education.

The results reveal nuanced mediation patterns that illuminate the mechanisms through which pre-service teachers’ attitudes toward ICT-enhanced teaching are formed. Socially interactive strategies, exemplified by Role Models and Collaboration, exerted particularly strong effects through perceived ease of use (PEOU), suggesting that observing exemplary peers and participating in cooperative learning primarily enhance teachers’ operational confidence while alleviating technology-related anxiety. The role models effect operates by allowing pre-service teachers to witness the practical implementation of ICT in authentic teaching contexts, transforming abstract or complex technological practices into concrete, replicable strategies (Tezci, 2011). By seeing how experienced peers integrate tools seamlessly into their instructional design, pre-service teachers gain clearer mental models of how to perform these tasks themselves, which reduces perceived effort and uncertainty (Habibi et al., 2022). Moreover, observing the tangible benefits of these practices—such as improved student engagement or more efficient lesson delivery—helps teachers recognize the functional value of ICT in supporting pedagogical goals (Joo et al., 2018). Concurrently, collaborative activities provide opportunities for peers to exchange strategies, co-construct solutions, and offer immediate feedback, facilitating the acquisition of procedural knowledge within realistic teaching scenarios (Barak, 2014). Collectively, these socially embedded experiences lower perceived complexity, foster a sense of mastery, and cultivate a shared professional culture that normalizes ICT integration in STEM education, thereby supporting both operational confidence and an appreciation of its educational utility (Kartal & Tasdemir, 2021).

Cognitive design strategies, including Reflection and Instructional Design, were more strongly associated with PU, underscoring their capacity to enhance teachers’ recognition of the pedagogical value of ICT. Structured reflection prompts pre-service teachers to critically examine their instructional approaches, evaluate the alignment between technology use and learning objectives, and anticipate potential challenges in implementation (Awad, 2018; Şenyiğit & Serin, 2022; B. Wu et al., 2021). Instructional design exercises, in turn, require systematic planning, sequencing of activities, and deliberate integration of ICT tools into lesson structures, providing concrete evidence of how technology can support and extend student learning (Barak, 2014; B. Wu et al., 2021; Galadima et al., 2019). These strategies enhance cognitive engagement by prompting pre-service teachers to critically evaluate the instructional value of ICT, assess its alignment with learning objectives, and explore its potential to improve conceptual understanding, problem-solving, and inquiry-based learning. By shifting the focus from the novelty of technology to its pedagogical utility, reflection and instructional design strengthen teachers’ ability to integrate ICT in ways that meaningfully advance student learning outcomes.

Experiential feedback strategies, namely Authentic Experience and formative Feedback, demonstrated balanced mediation effects through both PEOU and PU, highlighting their integrative role in teacher preparation. Authentic practice situates learning in real-world contexts, allowing pre-service teachers to bridge the gap between simulated training and the complex realities of classroom instruction (Avsec & Jagiełło-Kowalczyk, 2018; Valtonen et al., 2015). Such immersive experiences not only refine technical skills but also illustrate the situational relevance of ICT, thereby enhancing both operational ease and perceived instructional benefit (Barak, 2014; B. Wu et al., 2021). Complementing this, formative feedback provides timely, targeted input that enables teachers to refine their practice, correct misconceptions, and reinforce effective strategies (Şenyiğit & Serin, 2022; Yusuf et al., 2017). By combining hands-on application with continuous evaluative support, these strategies create a cyclical process of skill development and pedagogical refinement, ensuring that pre-service teachers emerge from training with both the competence and the confidence to integrate ICT in ways that are sustainable, contextually relevant, and instructionally impactful.

These differentiated patterns of influence suggest that the explanatory power of the TAM may be context-dependent, particularly for pre-service STEM teachers. Unlike general teacher populations, STEM teachers are characterized by cross-disciplinary integration, practice-oriented tasks, and a strong reliance on technology, which makes their perceptions of ease of use and usefulness potentially more sensitive to structured training interventions rather than solely individual experience (Venkatesh et al., 2002). While TAM traditionally emphasizes individual cognition, the findings indicate that professional learning environments and program design play a critical role in shaping the psychological pathways to technology adoption within this specific teacher population.

Building on the quantitative findings that link the six SQD strategies to teachers’ perceptions of ease of use and usefulness, the qualitative data collected in this study may also reflect certain aspects of the SQD2 model. For example, broad strategies such as Role Models, Collaboration, and Reflection could correspond to more specific subcomponents, including pedagogical approaches, group work, and technology scaffolding. The findings suggest that these elements might interact in ways that simultaneously support pre-service STEM teachers’ technical competence and pedagogical confidence. Further, these observations provide preliminary insights into how detailed and interactive strategies could function in practice, offering a basis for future research to systematically examine the mechanisms and potential synergies among SQD2 components.

From a practical perspective, the findings suggest that effective pre-service teacher preparation should adopt a strategically sequenced blend of these strategies (Tondeur et al., 2016; Tondeur et al., 2025). Early in training, role models experiences and collaboration can establish a foundation of confidence and operational ease; mid-stage activities, such as reflective practice and instructional design, can deepen pedagogical understanding; and culminating experiences that combine authentic practice with systematic feedback can consolidate both skill mastery and instructional alignment. Such a scaffolded approach ensures that novice STEM teachers enter the profession with not only the ability to operate ICT tools but also the capacity to deploy them in ways that meaningfully advance STEM learning objectives. This is particularly significant in China’s current education reform context, where the demand for inquiry-based, interdisciplinary STEM teaching continues to grow.

Beyond addressing the practical design of pre-service teacher education, this study advances theoretical understanding in several keyways. First, it focuses specifically on pre-service STEM teachers, whose teaching practices are more tightly integrated with ICT than those of general subject teachers. This population may exhibit unique patterns in the development of ICT-related teaching attitudes, and the study provides empirical support for how different pre-service training strategies selectively influence their attitudes. Second, the study responds to the contextual challenges in China, where a clear supply-demand gap exists in STEM teacher preparation. Despite the recognized importance of ICT teaching attitudes for shaping instructional behavior, their formation and development are constrained by limited pre-service training, scarce practical opportunities, and variability in educational support. By introducing the SQD model and integrating it with the TAM framework in a localized context, this research not only generates empirical evidence but also offers guidance for designing systematic and context-sensitive pre-service programs that effectively cultivate STEM teachers’ attitudes toward ICT-enhanced teaching. Moreover, the findings contribute preliminary evidence toward the evolution of the SQD model into the more detailed and interactive SQD2 framework. Finally, methodologically, the study combines mediation analysis with qualitative interviews, cross validating the influence paths of each SQD strategy on TAM constructs and providing a nuanced explanation of the mechanisms through which pre-service training shapes STEM teachers’ attitudes toward ICT-enhanced teaching.

## 6. Conclusions

In conclusion, this study provides empirical evidence that pre-service training strategies shape STEM teachers’ attitudes toward ICT-enhanced teaching through differentiated pathways. Socially interactive strategies mainly enhance perceived ease of use, cognitive design strategies strengthen perceived usefulness, and experiential feedback strategies support both dimensions, collectively fostering positive attitudes that facilitate sustainable ICT integration. By integrating the SQD model with the TAM, the study demonstrates the value of targeted program design in cultivating both technical competence and pedagogical confidence, particularly within the context of Chinese STEM teacher education.

Several limitations should be noted. The reliance on self-reported, cross-sectional data constrains causal inference, and the geographically concentrated sample limits generalizability. Future quantitative studies with larger samples could employ structural equation modeling (SEM) to examine the differentiated and interactive effects of training strategies on STEM teachers’ ICT attitudes more rigorously.

Future research could also further investigate how SQD strategies, including the interactive components emphasized in SQD2, function specifically for STEM teachers and how these micro-level strategies interact with meso- and macro-level factors, such as school culture, policy support, and curriculum context. Qualitative and intervention studies can provide deeper insight into STEM teachers’ lived experiences and the effectiveness of strategically sequenced programs in enhancing both perceived ease of use and perceived usefulness.

Overall, carefully designed pre-service training is essential for preparing STEM teachers’ attitudes toward ICT-enhanced teaching who are not only technically proficient but also able to integrate ICT meaningfully into instruction, promote inquiry-based learning, and support student success in increasingly digitalized STEM classrooms.

## Figures and Tables

**Table 1:** Descriptive Statistics of Early-Career STEM Teachers’ ICT Attitudes.

	N	Min	Max	Mean	SD
PU	309	3.00	5.00	4.28	0.41
PEOU	309	3.00	5.00	4.25	0.42
ICT-ATT	309	3.20	5.00	4.26	0.37

**Table 2:** Descriptive Statistics of Pre-Service Training Strategy Implementation.

	N	Min	Max	Mean	SD
Role Models (ROL)	309	3.00	5.00	4.20	0.38
Collaboration (COL)	309	3.00	5.00	4.20	0.43
Reflection (REF)	309	3.00	5.00	4.18	0.43
Instructional Design (DES)	309	2.00	5.00	4.14	0.52
Authentic Experience (AUT)	309	3.00	5.00	4.24	0.40
Feedback (FEE)	309	1.75	5.00	4.01	0.64

**Table 3:** The correlation between pre-service training strategies and STEM teachers’ attitude.

	Gender	Age	EB	Subject	Grade	TA	ICT-ATT	PU	PEOU	ROL	COL	REF	DES	AUT	FEE
Gender	1	−0.206 **	0.124 **	0.292 **	−0.288 **	−0.228 **	0.058 **	−0.025 **	−0.081 **	0.104 **	−0.008 **	−0.042 **	−0.017 *	−0.018 **	−0.099 **
Age		1	0.207 **	−0.256 **	0.261 **	0.510 **	−0.001 **	0.080 **	0.051 **	−0.081 **	−0.061 **	0.156 **	0.013 **	−0.021 **	0.074 **
EB			1	−0.063 **	0.060 **	0.019 **	0.134 **	0.046 **	0.188 **	0.220 **	0.089 **	0.145 **	0.040 **	0.067 **	0.053 **
Subject				1	−0.361 **	−0.067 **	−0.033 **	−0.058 **	−0.053 **	−0.098 **	−0.084 **	−0.098 **	−0.173 **	−0.033 **	−0.086 **
Grade					1	0.315 **	0.034 **	0.178 **	0.072 **	0.090 **	0.059 **	0.151 **	0.103 **	0.120 **	0.086 **
TA						1	0.055 **	0.190 **	0.127 **	0.026 **	0.050 **	0.137 **	0.127 **	0.161 **	0.156 **
ICT-ATT							1	0.611 ***	0.677 ***	0.554 **	0.584 ***	0.467 **	0.478 **	0.513 **	0.399 **
PU								1	0.577 ***	0.378 **	0.474 **	0.476 **	0.500 **	0.456 **	0.487 **
PEOU									1	0.420 **	0.418 **	0.347 **	0.345 **	0.438 **	0.346 **
ROL										1	0.540 **	0.454 **	0.444 **	0.487 **	0.245 **
COL											1	0.459 **	0.691 **	0.541 **	0.494 **
REF												1	0.566 **	0.536 **	0.529 **
DES													1	0.594 **	0.618 **
AUT														1	0.526 **
FEE															1

Note: * *p* < 0.05, ** *p* < 0.01, *** *p* < 0.001. EB: Educational Background; TA: Teaching Age; ICT-ATT: General attitudes toward ICT in STEM education; PEOU: Perceived Ease of Use; PU: Perceived Usefulness; ROL: Role Models; COL: Collaboration; REF: Reflection; DES: Instructional Design; AUT: Authentic Experience: FEE: Feedback.

**Table 4:** The mediating effect of PEOU and PU for ROL.

		Effect	Se	LLCI	ULCI	Effect Size
PEOU	Total Effect	0.53	0.05	0.44	0.62	
	Direct Effect	0.30	0.04	0.22	0.38	56.60%
	Indirect Effect	0.23	0.03	0.17	0.31	43.40%
PU	Total Effect	0.53	0.05	0.44	0.62	
	Direct Effect	0.33	0.04	0.25	0.41	62.26%
	Indirect Effect	0.20	0.03	0.14	0.27	37.74%

**Table 5:** The mediating effect of PEOU and PU for COL.

		Effect	Se	LLCI	ULCI	Effect Size
PEOU	Total Effect	0.53	0.04	0.46	0.60	
	Direct Effect	0.33	0.03	0.27	0.40	62.26%
	Indirect Effect	0.20	0.03	0.15	0.25	37.74%
PU	Total Effect	0.53	0.04	0.46	0.60	
	Direct Effect	0.33	0.04	0.26	0.41	62.26%
	Indirect Effect	0.20	0.03	0.14	0.26	37.74%

**Table 6:** The mediating effect of PEOU and PU for REF.

		Effect	Se	LLCI	ULCI	Effect Size
PEOU	Total Effect	0.42	0.04	0.34	0.50	
	Direct Effect	0.23	0.03	0.16	0.30	54.76%
	Indirect Effect	0.19	0.03	0.14	0.25	45.24%
PU	Total Effect	0.42	0.04	0.34	0.50	
	Direct Effect	0.20	0.04	0.12	0.27	47.62%
	Indirect Effect	0.22	0.03	0.17	0.28	52.38%

**Table 7:** The mediating effect of PEOU and PU for DES.

		Effect	Se	LLCI	ULCI	Effect Size
PEOU	Total Effect	0.41	0.03	0.35	0.47	
	Direct Effect	0.26	0.03	0.20	0.31	63.41%
	Indirect Effect	0.15	0.02	0.11	0.20	36.59%
PU	Total Effect	0.41	0.03	0.35	0.47	
	Direct Effect	0.22	0.03	0.16	0.29	53.66%
	Indirect Effect	0.19	0.03	0.14	0.24	46.34%

**Table 8:** The mediating effect of PEOU and PU.

		Effect	Se	LLCI	ULCI	Effect Size
PEOU	Total Effect	0.51	0.04	0.43	0.59	
	Direct Effect	0.26	0.04	0.18	0.34	50.98%
	Indirect Effect	0.25	0.03	0.19	0.31	49.02%
PU	Total Effect	0.51	0.04	0.43	0.59	
	Direct Effect	0.29	0.04	0.21	0.37	56.86%
	Indirect Effect	0.22	0.03	0.16	0.28	43.14%

**Table 9:** The mediating effect of PEOU and PU.

		Effect	Se	LLCI	ULCI	Effect Size
PEOU	Total Effect	0.28	0.03	0.23	0.34	
	Direct Effect	0.15	0.02	0.10	0.20	53.57%
	Indirect Effect	0.13	0.02	0.10	0.17	46.43%
PU	Total Effect	0.28	0.03	0.23	0.34	
	Direct Effect	0.12	0.03	0.07	0.18	53.57%
	Indirect Effect	0.16	0.02	0.12	0.20	46.43%

## Data Availability

All survey data are available from the corresponding author upon request. The data that support the findings of this study are available from Shanghai Jiao Tong University, but restrictions apply to the availability of these data, which were used under license for the current study and so are not publicly available. The data are, however, available from the corresponding author upon reasonable request.

## Appendix A

**Table A1:** Full Measurement Items in English and Chinese.

	Constructs	Items	Source
Attitude	General attitudes toward ICT-enhanced teaching (ICT-ATT)	ICT-ATT1: The use of ICT is useful to me. 信息技术的使用是非常有用的。	Scherer et al. (2018)
		ICT-ATT2: Working with ICT is very interesting to me. 利用信息技术进行工作是非常有趣的。	
		ICT-ATT3: I would like to know a lot about ICT. 了解更多关于信息技术的知识是有必要的。	
		ICT-ATT4: I think it’s important to be able to use ICT. 能够熟练使用信息技术是很重要的。	
		ICT-ATT5: I like talking to others about ICT. 我经常和别人谈论信息技术。	
	Perceived Ease of Use (PEOU)	PEOU1: I can easily find the medical services or doctors I need on this platform. 信息技术的使用是比较容易的。	
		PEOU2: I think it is simple to make appointments or consult through this platform. 熟练掌握信息技术的使用方法是比较容易的。	
		PEOU3: I think the entire process of using this platform is quick and convenient. 学习使用信息技术进行工作是比较容易的。	
	Perceived Usefulness (PU)	PU1: The use of ICT allows me to prepare my lessons faster. 信息技术的使用让我能够更快地备课。	
		PU2: The use of ICT allows me to prepare my classes better. 信息技术的使用让我能够更好地备课。	
		PU3: I find ICT useful for my work as a teacher. 信息技术对我作为一名教师的工作是很有用的。	
Training Strategies	ROL	ROL1: I saw many examples of ICT use in an educational setting. 我看到很多同行在教学中使用信息技术的例子。	Tondeur et al. (2016)
		ROL2: I observed sufficient ICT use in an educational setting in order to integrate applications myself in the future. 我会通过观察同行的信息化教学行为来提升自己的信息化教学能力。	
		ROL3: I saw good examples of ICT practice that inspired me to use ICT applications in the classroom myself. 同行信息化教学的成功案例会促进我在课堂教学中对信息技术的使用。	
		ROL4: The potential of ICT use in education was demonstrated concretely. 同行的信息化教学行为能够很好地展现信息技术在教育中的巨大潜力。	
	COL	COL1: There were enough occasions for me to work together with other students on ICT use in education (i.e., we developed ICT-based lessons together). 我有很多机会可以和同行一起利用信息技术进行教学（如，我们可以一起开发基于信息技术的教学课程）。	
		COL2: I was convinced of the importance of co-operation with respect to ICT use in education. 在利用信息技术进行教学时，同行之间的合作是非常重要的。	
		COL3: Students helped each other to use ICT in an educational context. 我和同行在利用信息技术进行教学时会互相帮助。	
		COL4: Experiences using ICT in education were shared. 我和同行会分享在教学中使用信息技术的经验。	
	REF	REF1: I was given the chance to reflect on the role of ICT in education. 我经常思考信息技术对教育的作用。	
		REF2: We discussed the challenges of integrating ICT in education. 我和同行会讨论将信息技术纳入教育时所面临的挑战。	
		REF3: We were given the opportunity to discuss our experiences with ICT in the classroom (i.e., during internships). 我和同行会在课堂上讨论使用信息技术进行教学的相关经验。	
		REF4: There were specific occasions for us to discuss our general attitude towards ICT in education. 我和同行会对大家在教学中使用信息技术的态度进行反思和讨论。	
	DES	DES1: I received sufficient help in designing lessons that integrated ICT. 在设计整合信息技术的课程时，我得到了足够多的帮助。	
		DES2: We learnt how to thoroughly integrate ICT into lessons. 我能熟练地将信息技术融入教学课程。	
		DES3: We received help to use ICT when developing educational material. 在利用信息技术研制教学材料时，我得到了足够多的帮助。	
		DES4: I received a great deal of help developing ICT-rich lessons and projects to use for my internship. 实习实践期间开发富含信息技术的课程和项目时，我得到了足够多帮助。	
	AUT	AUT1: There were enough occasions for me to test different ways of using ICT in the classroom. 我有足够多的机会在课堂上对信息技术的使用方式进行测试。	
		AUT2: I was encouraged to gain experience in using ICT in a classroom setting. 我被鼓励在课堂实践中积累信息化教学的相关经验。	
		AUT3: Students were encouraged when they attempted to use ICT in an educational setting. 我被鼓励在教学中使用信息技术。	
	FEE	FEE1: I received sufficient feedback about the use of ICT in my lesson. 我收到了很多关于我信息化教学行为的反馈和评价。	
		FEE2: I received sufficient feedback on how I can further develop my ICT competence. 我收到了很多关于如何进一步提升自己信息化教学能力的建议。	
		FEE3: My competences in using ICT in the classroom were regularly evaluated. 我的信息化教学能力得到了定期评估。	
